# Analysis of hospital and payer costs of care: aggressive warming versus routine warming in abdominal major surgery

**DOI:** 10.3389/fpubh.2023.1256254

**Published:** 2023-11-02

**Authors:** Shujia Song, Lijian Pei, Hongda Chen, Yuelun Zhang, Chen Sun, Jie Yi, Yuguang Huang

**Affiliations:** ^1^Department of Anesthesiology, Peking Union Medical College Hospital, Chinese Academy of Medical Sciences and Peking Union Medical College, Beijing, China; ^2^Institute for Clinical Medical Research, Peking Union Medical College Hospital, Chinese Academy of Medical Sciences and Peking Union Medical College, Beijing, China

**Keywords:** hypothermia, cost–benefit analysis, randomized controlled trials as topic, time factors, hospital costs

## Abstract

**Background:**

Hypothermia is common and active warming is recommended in major surgery. The potential effect on hospitals and payer costs of aggressive warming to a core temperature target of 37°C is poorly understood.

**Methods:**

In this sub-analysis of the PROTECT trial (clinicaltrials.gov, NCT03111875), we included patients who underwent radical procedures of colorectal cancer and were randomly assigned to aggressive warming or routine warming. Perioperative outcomes, operation room (OR) scheduling process, internal cost accounting data from the China Statistical yearbook (2022), and price lists of medical and health institutions in Beijing were examined. A discrete event simulation (DES) model was established to compare OR efficiency using aggressive warming or routine warming in 3 months. We report base-case net costs and sensitivity analyses of intraoperative aggressive warming compared with routine warming. Costs were calculated in 2022 using US dollars (USD).

**Results:**

Data from 309 patients were analyzed. The aggressive warming group comprised 161 patients and the routine warming group comprised 148 patients. Compared to routine warming, there were no differences in the incidence of postoperative complications and total hospitalization costs of patients with aggressive warming. The potential benefit of aggressive warming was in the reduced extubation time (7.96 ± 4.33 min vs. 10.33 ± 5.87 min, *p* < 0.001), lower incidence of prolonged extubation (5.6% vs. 13.9%, *p* = 0.017), and decreased staff costs. In the DES model, there is no add-on or cancelation of operations performed within 3 months. The net hospital costs related to aggressive warming were higher than those related to routine warming in one operation (138.11 USD vs. 72.34 USD). Aggressive warming will have an economic benefit when the OR staff cost is higher than 2.37 USD/min/person, or the cost of disposable forced-air warming (FAW) is less than 12.88 USD/piece.

**Conclusion:**

Despite improving OR efficiency, the economic benefits of aggressive warming are influenced by staff costs and the cost of FAW, which vary from different regions and countries.

**Clinical trial registration:**

clinicaltrials.gov, identifier (NCT03111875).

## 1. Introduction

Perioperative hypothermia, which is defined as a body temperature of less than 36°C (1), is one of the most common complications for surgical patients. It was (2) reported that the incidence of intraoperative hypothermia is 40% in China, and only 10.7% of patients received aggressive warming during surgery. Considering the complications caused by perioperative hypothermia, including coagulopathy (3), surgical site infection (4), and prolonged recovery (5–8), intraoperative thermal management has been advocated for. However, despite broad beliefs and guideline recommendations (9–11), it is controversial to maintain normothermia near 37°C using aggressive warming. Recently, an international multicenter randomized trial of 5,056 patients undergoing non-cardiac surgery, PROTECT, reported that the incidence of major cardiovascular events, surgical site infections, and transfusion requirement did not differ in patients randomly allocated to 35.5°C and 37°C (12).

Considering the high cost of aggressive warming due to special equipment and disposable forced-air warming (FAW) blankets (13), it is necessary to evaluate the economic benefits of intraoperative aggressive warming. According to recent studies, the total hospitalization expenditure is highly related to postoperative complications from the perspective of patients (14). In hospitals, intraoperative hypothermia could prolong recovery time (6–8), whereas aggressive warming could decrease recovery time (5), which could result in enhanced operation room (OR) efficiency and decreased staff costs.

This study aimed to determine whether aggressive warming can enhance operating room efficiency and provide economic benefits for patients and hospitals.

## 2. Materials and methods

### 2.1. Study population and data collection

This study used data from the PROTECT trial (clinicaltrials.gov, NCT03111875) in which patients were randomly assigned to the aggressive or routine warming group. The original randomized controlled trial was performed in compliance with the Declaration of Helsinki, approved by the Ethics Committees of Peking Union Medical College Hospital (PUMCH). Patients in the aggressive warming group were warmed to an intraoperative core temperature of 37°C during surgery with FAW blankets. In contrast, in the routine warming group, quilts were used for a target temperature of 35.5°C, and warming did not begin until the core temperature was below 35.5°C (12).

In this study, we included 309 patients who received elective abdominal surgery between May 18, 2016, and December 3, 2020, in the PUMCH PROTECT trial, excluding 124 patients whose final core temperature was between 35.8 and 37.7°C, and 431 patients who were transferred to the ICU after surgery with tracheal intubation. The inclusion criteria for PROTECT were also applicable, including age ≥ 45 years, having at least one cardiovascular risk factor, and receiving major non-cardiac surgery that lasted between 2 to 6 h under general anesthesia. Patients were excluded if they had coagulopathy, end-stage renal disease requiring dialysis, body mass index exceeding 30 kg/m^2^, or if they were not extubated in the OR. Patients were divided into the aggressive warming group and the routine warming group according to their final core temperature (≥ 36.8°C or ≤ 35.7°C).

The baseline data collected from the Anesthesia Information Management Systems (AIMS) included the duration of the procedure (time from OR admission to the end of surgery), extubation time (time from the end of surgery to extubation), turnover time (time from extubation to the admission for the next procedure in the same OR), and the incidence of prolonged extubation. Prolonged extubation is defined as a 15-min or longer interval from the end of surgery to the removal of the tracheal tube (15, 16). The effects of aggressive warming on OR efficiency include extubation time, incidence of prolonged extubation, and turnover time.

## 3. Costs estimates

### 3.1. Staff costs

It is assumed that when an OR runs for over 16:00, surgeons, anesthesiologists, and nurses in the OR and post anesthesia care unit (PACU) are eligible for overtime pay. Estimates of OR staff costs in hospitals in China are limited. The average annual salary of health practitioners in Beijing in 2022 according to the China Statistical Yearbook 2022^1^ was converted to United States dollars (USD) using the average exchange rate in 2022 (1 USD = 6.7261 China Yuan). Assuming the working time was 8 h/day, 250 working days/year, and there were 2 surgeons, 2 anesthesiologists, and 2 nurses in an OR room, and 2 anesthesiologists and 2 nurses in PACU, the time saved in an OR has a value of 0.27 USD/min/person.

### 3.2. Costs of equipment in thermal management

Patients in the routine warming group were warmed using a quilt at 4.46 USD per piece. For aggressive warming, equipment costs were 78.68 USD, including direct and indirect costs. The details are provided in Supplementary Table S1.

### 3.3. Direct costs for the aggressive warming

(1) Medical supplies: Imported disposable forced-air warming blanket (3 M™ Bair Hugger™ 635) was priced at 74.34 USD per piece, and was acquired directly from the 3 M company and (2) Energy cost: Considering the rated power and usage time, the electricity bill for the forced-air warming system was 0.3 USD/h.

### 3.4. Indirect costs for the aggressive warming

(1) Medical equipment depreciation: An FAW blanket with a warming unit (3 M Bair Hugger 700) was used. The air compressor was assumed to have a depreciation period of 5 years, with an average of 1,031 radical operations for elective colorectal cancer in a year. The cost of equipment depreciation was estimated at 2.88 USD/operation and (2) Repair and maintenance: The replacement filter should be changed every 12 months to maintain the maximum efficiency of the warming unit. The maintenance cost for the FAW system was estimated to be 0.43 USD/operation.

## 4. Discrete event simulation (DES) model

**Figure 1:**
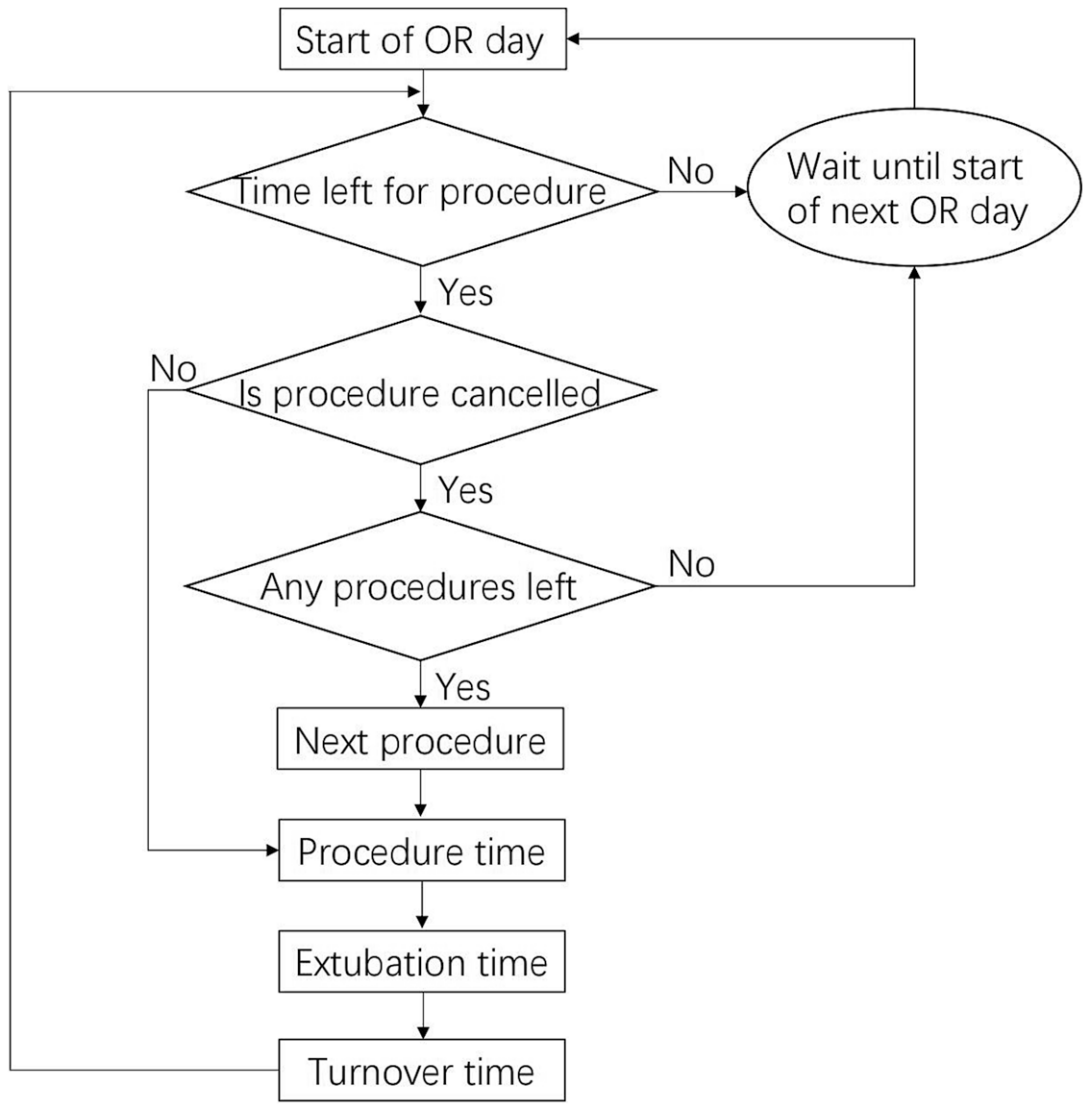
Diagram of procedural flow in an operating room.

**Figure 2:**
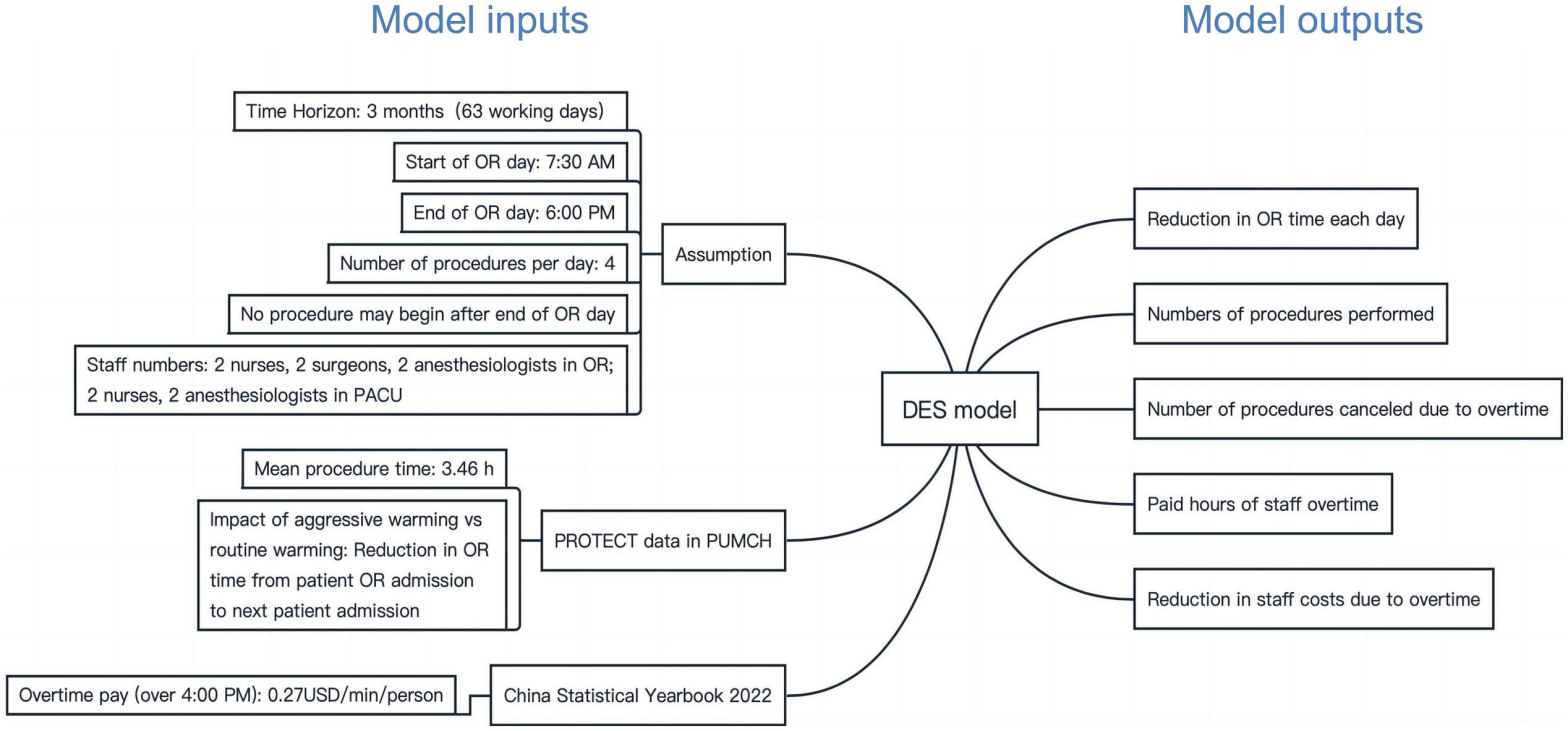
Input and output of the DES model for OR efficiency in 3 months.

The DES model was developed using R Studio software (version 1.1.463). The model compares OR efficiency using aggressive warming or routine warming for the same simulated schedule in an OR on 63 working days over 3 months in a hypothetical hospital of the same size as PUMCH in China. The procedure flow on the day of the operation is shown in Figure 1. The parameters influencing the procedure flow included the start time of the OR, end time of the OR, mean procedure time, number of procedures per day, cancelation policy, the impact of aggressive warming in OR time, staff numbers and overtime pay, which are described with data sources in Figure 2. The outputs of the DES model include the number of procedures performed, number of procedures canceled owing to overtime, paid hours of staff overtime, and a reduction in staff costs owing to overtime (Figure 2).

## 5. Economic analyses

### 5.1. Base-case cost analysis

Charges to patient for thermal management included temperature monitoring (3.12 USD/h. Less than one hour was charged for one hour) and aggressive warming therapy (2.97 USD/h. Less than one hour was charged for one hour).

From the perspective of the hospital, the net costs related to thermal management were analyzed, which were the sum of the equipment and staff costs in the base-case scenario. Equipment costs were analyzed considering that the FAW was used with an air compressor for aggressive warming, while the quilt was used for routine warming.

Staff costs were treated as variables with more than 8 h of workload. Reducing OR time reduces direct staff costs when operating rooms are used during overtime. In our analysis, the OR ran for over 8 h on each working day, which is consistent with reality. Using a previously reported method (17), staff costs related to OR time were calculated by multiplying the annual salary of each staff member on a per-minute basis by the number of staff and the length of extubation and turnover time.

### 5.2. Sensitivity analysis

Sensitivity analysis was conducted to determine the impact of the independent variables on the net hospital costs of thermal management. Variables, including procedure time, staff costs, and FAW costs, varied widely and were incorporated into separate sensitivity analyses. The pair-wise threshold analysis that varied FAW and staff costs as well as the reduction in OR time and staff costs can provide generic information that can be adjusted to the specific circumstances of individual regions and hospitals.

## 6 Statistical analyses

Continuous variables are presented as mean and standard deviation (SD) or median (25–75 IQR), while categorical variables are reported as numbers of patients and percentages. For economic analysis, data had to be included as arithmetic rather than geometric means (17, 18). Differences in categorical variables between aggressive and routine warming were tested using the χ2 test or Fisher’s exact test. *p* < 0.05 was considered statistically significant. Statistical analyses were performed using SPSS software v.21.0 (IBM SPSS Statistics, IBM Corporation, NY, United States).

## 7. Results

### 7.1. Baseline characteristics and perioperative outcomes

In total, 309 patients from the PROTECT trial were included in this study, of whom 148 underwent routine warming and 161 underwent aggressive warming. The demographics and preoperative characters were similar (Table 1).

**Table 1:** Baseline characteristics of patients.

	Routine warming (*n* = 148)	Aggressive warming (*n* = 161)	*p* value
Age(y)	67 ± 6	67 ± 7	0.421
Male sex	105(71)	121(75)	0.695
BMI (kg/m2)	23 ± 3	24 ± 3	0.545
ASA			0.719
I	2(1.3)	4(2.5)	
II	96(64.9)	99(61.5)	
III	50(33.8)	58(36)	
Comorbidity			
HTN	85(57)	75(47)	0.057
DM	39(26)	37(23)	0.492
CAD	15(10)	18(11)	0.766
CRF	2(1)	1(1)	0.942
PAD	4(3)	10(6)	0.139
COPD	3(2)	2(1)	0.924
Current smoking	51(35)	54(34)	0.865

Results presented as x ± s or n (%).

BMI, body mass index; HTN, hypertension; DM, diabetes mellitus; CRF, chronic renal failure; CAD, coronary artery disease; COPD, chronic obstructive pulmonary disease; PAD, peripheral arterial disease.

Patients in the aggressive warming group had a mean final core temperature of 37.0 ± 0.2°C, and 35.4 ± 0.2°C in the routine warming group. There was no significant difference in procedure time, intraoperative blood loss, major postoperative complications, length of stay (LOS), and hospitalization costs for patients between groups. No patient in the routine warming group required rescue warming. The reduction in extubation time was 2.37 min (aggressive warming vs. routine warming, 7.96 ± 0.34 min vs. 10.33 ± 0.48 min, *p* < 0.001), while the reduction in turnover time was 0.76 min without a statistical difference (aggressive warming vs. routine warming, 14.05 ± 0.90 min vs. 14.81 ± 0.92 min, *p* = 0.553). Consequently, the reduction in OR time, which included a reduction in extubation time and turnover time was 3.13 min per procedure in the aggressive warming group (aggressive warming vs. routine warming, 22.01 ± 10.28 min vs. 25.14 ± 10.02 min, *p* = 0.010). Significantly fewer patients in the aggressive warming group suffered from prolonged extubation than in the routine warming group (aggressive warming vs. routine warming, 5.6% vs. 13.5%, *p* = 0.017). The main perioperative outcomes are summarized in Table 2.

**Table 2:** Perioperative outcomes.

	Routine warming (*n* = 148)	Aggressive warming (*n* = 161)	*p* value
Procedure time^a^ (h)	3.31 ± 0.98	3.60 ± 1.18	0.058
Mean final core temperature (°C)	35.4 ± 0.2	37.0 ± 0.2	0.005
Operative blood loss (mL)	76.35 ± 102.63	87.98 ± 146.90	0.424
Postoperative complications			
SSI	15(10.1)	11(6.8)	0.296
HAI	24(16.2)	23(14.3)	0.637
MINS	0	0	-
Length of stay (LOS)	11(10, 14)	11(10, 14)	0.866
Total hospitalization costs for patients (USD)	9515.17 ± 9916.59	8132.5 ± 4742.72	0.483
Extubation time^b^ (min)	10.33 ± 5.87	7.96 ± 4.33	<0.001
Prolonged extubation	20 (13.5)	9 (5.6)	0.017
Turnover time^c^ (min)	14.81 ± 9.54	14.05 ± 9.47	0.553
The sum of extubation and turnover time	25.14 ± 10.02	22.01 ± 10.28	0.010

Values were expressed as Mean ± SD or median (Q1, Q3) or n (%).

a Procedure time = interval from the patient’s entry into the OR until the end of surgery (i.e., dressing on).

b Extubation time, time from end of surgery to extubation.

c Turnover time, time from extubation to the admission of the next procedure or the off-time of OR staff.

SSI, organ/space surgical site infection; HAI, hospital-acquired Infection; MINS, myocardial injury after non-cardiac surgery.

### 7.2. Or efficiency over 3 months in DES model

The DES model outputs reported OR efficiency with aggressive warming and routine warming in the OR over 3 months (Table 3). Compared to routine warming, there was no change in the number of operations canceled owing to overtime or the total number of procedures performed with aggressive warming. With the use of the disposable FAW for aggressive warming, staff costs due to overtime were reduced by 1591.03 USD over 3 months saving an average of 9.39 min each OR day.

**Table 3:** DES model: OR efficiency over 3 months.

OR efficiency outcomes	Routine warming	Aggressive warming
Reduction in OR time each day (min)	-	9.39
Number of procedures performed	188	188
Number of procedures canceled due to overtime	63	63
Paid hours of staff overtime (hrs)	197.6	187.8
Reduction in staff costs due to overtime (USD)	-	1591.03

OR, operation room.

### 7.3. Base-case cost analysis

The charges to the patients related to thermal management included an FAW blanket, temperature monitoring, and aggressive warming therapy (Table 4). Patients who underwent aggressive warming were required to pay 98.7 USD for thermal management and patients who underwent routine warming were required to pay 12.48 USD.

**Table 4:** Base case costs of thermal management in one operation.

Costs (USD)	Routine warming	Aggressive warming
Charges to patients*		
FAW blanket	0	74.34
Temperature monitoring	12.48	12.48
Aggressive warming therapy	0	11.88
Overall	12.48	98.7
Hospital costs		
Equipment costs	4.46	78.68
Staff costs	67.88	59.43
Net costs	72.34	138.11

*Charges to patients: Patient charges related to thermal management, including temperature monitoring (3.12 USD/h) and aggressive warming therapy (2.97 USD/h), were obtained from the Beijing Medical Service Charging Standard. Less than one hour was charged for one hour.

Forced-air warming, FAW.

The net costs of each procedure for the base-case analysis from a hospital perspective are outlined in Table 4. The net costs of aggressive warming were 138.11 USD, which were 72.34 USD with routine warming. The savings in staff costs owing to a reduction in OR time did not cover the higher equipment costs of FAW.

### 7.4. Sensitivity analysis

#### 7.4.1. Procedure time

Considering that the next procedure could not be performed if it was completed after 18: 00, we performed a sensitivity analysis for changes in the procedure time. Only when the procedure time was between 153.24 min and 155.32 min, was it possible to accommodate 4 operations per day in an OR with aggressive warming, and 3 operations with routine warming. In other words, when the length of the procedure was maintained within this range, FAW was used to perform one more procedure than routine warming.

#### 7.4.2. Staff costs

**Figure 3:**
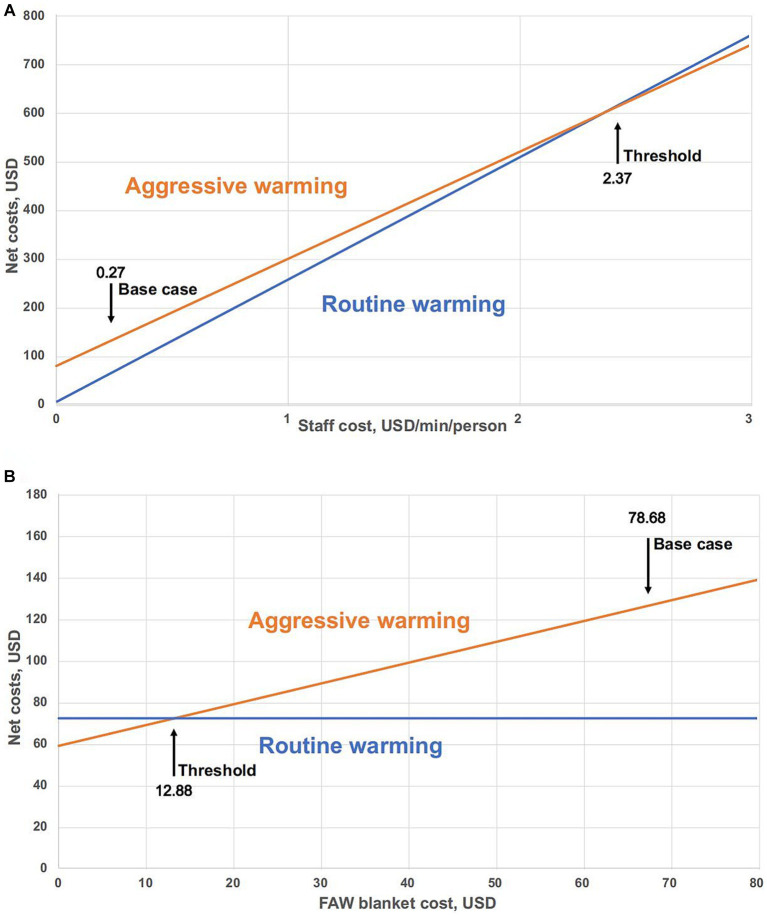
Threshold analysis of aggressive warming and routine warming. **(A)** Sensitivity analysis of net costs as a function of staff cost (USD/min/person). Base case cost is the value of staff cost used in the base-case analysis. Threshold: Aggressive warming versus routine warming = 2.37 USD/min/person. **(B)** Sensitivity analysis for net costs of varied FAW blanket in aggressive warming management. Threshold: point of equivalent net costs. Base case is the value of FAW blanket cost used in the base-case analysis. Threshold: Aggressive warming versus routine warming = 12.88 USD.

The staff costs per minute in an OR varies by region. The threshold value of staff costs was 2.37 USD/min/person (Figure 3A), at which point economic savings produced by reductions in OR time were equivalent to the higher equipment costs of aggressive warming. Another way of interpreting Figure 3A is that if the actual value of each staff member is 2.37 USD/min/person or higher, aggressive warming is cost-effective. At the base-case staff costs (0.27 USD/min), aggressive warming with disposable FAW blanket resulted in higher net costs than routine warming.

#### 7.4.3. FAW costs

With other factors unchanged, we conducted a sensitivity analysis on the change of FAW cost. The threshold for equivalent net costs between aggressive warming and routine warming is 12.88 USD (Figure 3B). Under the base-case scenario, the cost of FAW (78.68 USD) is much higher than the threshold, which means that aggressive warming is less economical at the current cost.

#### 7.4.4. Pair-wise threshold analysis of FAW cost and staff cost

**Figure 4:**
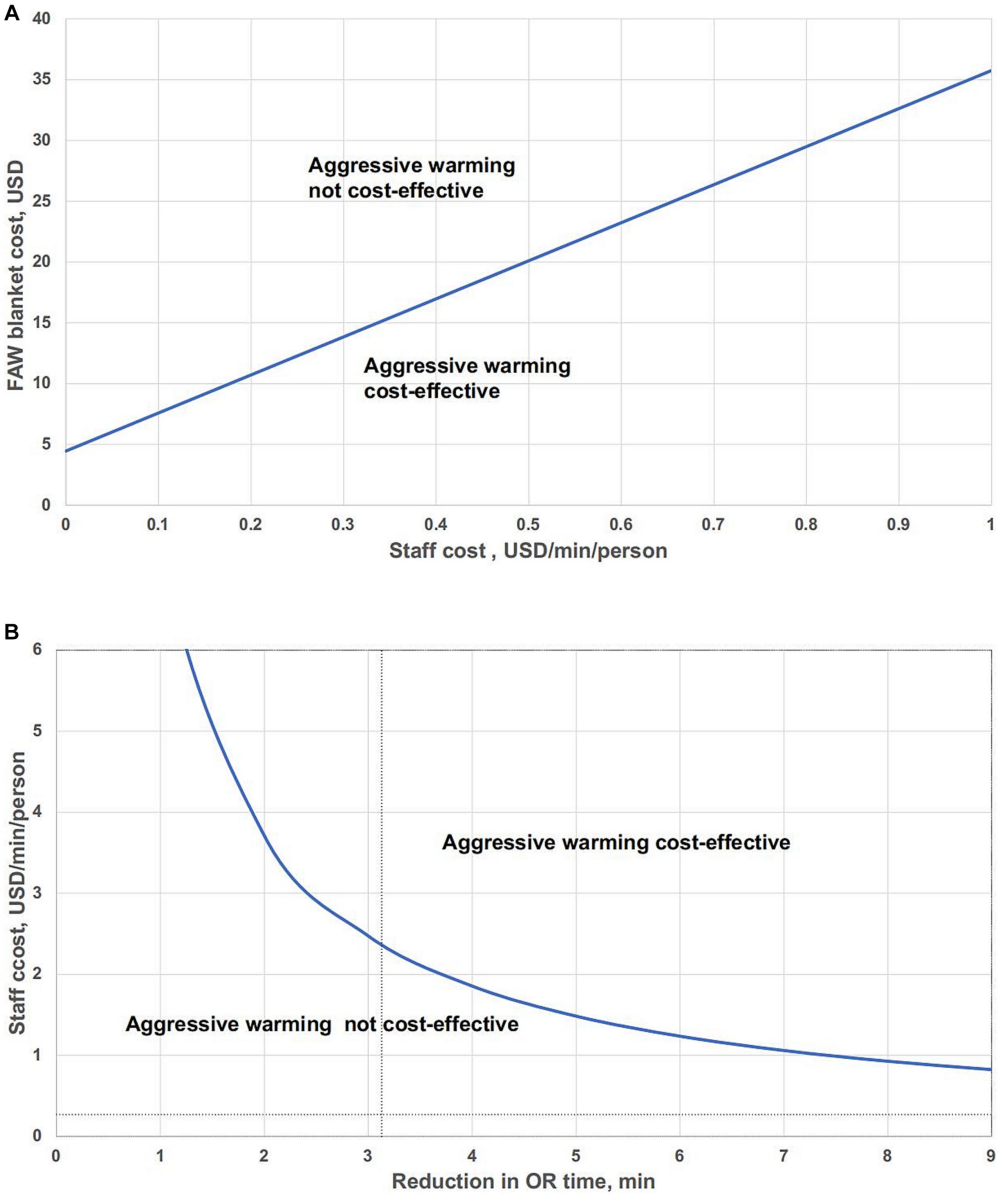
Pair-wise threshold analysis of the impact of variables in aggressive warming on the net costs. **(A)** Pair-wise threshold analysis of FAW blanket cost and staff costs. **(B)** Pair-wise threshold analysis: reduction in OR time vs. staff cost.

We conducted a pair-wise threshold analysis, primarily to explore, based on the current reduction in OR time, what the value of OR staff per minute is, and whether the cost of FAW is justified (Figure 4A). The linear equation suggests threshold values when net costs of these two thermal management systems are equivalent.


$$4.46+10\times \left(\mathrm{Staff}\;\mathrm{cost}\right)\times \left(3.13\right)=FAW\;\mathrm{costs}$$

Values to the lower right of the line indicate that aggressive warming is cost-effective; values to the upper left of the line suggest that aggressive warming is not cost-effective compared to routine warming.

#### 7.4.5. Pair-wise threshold analysis of reduction in OR time and staff cost

In this analysis, the curve represented staff cost per minute and reduction in OR time that differ over a range, with total costs equal for either aggressive warming or routine warming (Figure 4B). Values above the bold curve represent that aggressive warming is cost-effective and values below the curve mean that aggressive warming is not cost-effective. The intersection of the vertical dotted line and the horizontal dotted line represented the base-case assumptions, which is under the curve, indicating that aggressive warming is not cost-effective.

## 8. Discussion

In this study, we found no significant difference in postoperative complications and total hospitalization costs of patients with aggressive warming or routine warming. Although aggressive warming could reduce OR time (the sum of extubation time and turnover time) by 3.13 min, it did not result in additional operations in the DES model. Based on the current pricing policies, aggressive warming would be cost-effective if the healthcare system values the cost of each staff member to be at least 2.37 USD/min or the cost of FAW is to be less than 12.88 USD/piece.

From the perspective of the payer, there was no significant difference in the total hospital costs in our study, which is due to the lack of difference in the morbidity of postoperative complications (12, 14). Besides, with the development of laparoscopic surgery of colorectal cancer with little blood loss, few patients suffer hypothermia lower than 35.5°C if the ambient temperature is set appropriately (19). In consequence, whether aggressive warming to a target of 37°C can be economically beneficial from the perspective of a hospital is a matter of debate.

### 8.1. OR efficiency

We found that the increase of OR efficiency mainly originated from reduced extubation time. Our findings regarding OR efficiency were similar to those of Fleisher LA and colleagues (20), who assigned 100 patients undergoing elective surgery with FAW or routine thermal care with final core intraoperative temperatures of 35.4°C and 36.8°C, respectively. They reported that the time from the end of surgical dressing to extubation was reduced by 4 min in the FAW group. However, they only analyzed the association between potential savings from FAW and the percentage of variable components of intraoperative costs. A formal overall cost calculation was not performed in this study because accurate cost data were unavailable. In addition to the extubation time, the turnover time might be influenced by prolonged extubation (>15 min) intangibly because of the frustrated surgeons. Dexter F and colleagues (15) found that patients with prolonged extubation time (>15 min) caused an increased delay of 4.9 min longer between leaving OR and the next surgery beginning. Considering the incidence of prolonged extubation, they found that the turnover time may be increased by an average of 0.5 min in each procedure, which was similar to 0.76 min in our results. So, we took the reduction in both extubation time and turnover time into consideration due to aggressive warming.

Whether the marginal improvements in OR efficiency can be coupled with the potential benefits of accommodating additional operations or preventing operation cancelations caused by exceeding the allocated OR time should be considered. Insinga et al. (21) constructed a simulation model reflecting the enhanced OR efficiency and the prevention of procedure cancelations caused by overtime. They specifically focused on the use of sugammadex, which reduced recovery time by 14 min compared to neostigmine. However, few studies have investigated whether aggressive warming increases the number of procedures required. In our DES model, although the OR time was significantly reduced, it seems unlikely to perform one more procedure with such a 3.13 min reduction in OR time with aggressive warming, implying that the time savings could not lead to an improvement in OR productivity. The sensitivity analysis of procedure time demonstrated that aggressive warming would allow one more procedure if 153.24 min ≤ procedure time < 155.32 min. The timeframe was too narrow for anesthesiologists and surgeons to complete the induction and surgery within this range. In other words, small reductions in OR time due to aggressive warming did not permit an additional procedure to be performed.

### 8.2. Cost analysis

From the perspective of a hospital, besides OR efficiency, whether aggressive warming is cost-effective also depends on the costs of thermal management and the value of each minute of OR time saved.

The primary cost of aggressive warming originates from the FAW, which is the most commonly used and effective device to prevent perioperative hypothermia (22–25). We estimated the cost of FAW based on the price of the equipment sold by Bair Hugger in China and the cost of electricity, depreciation and repairs, which was 78.68 USD/piece. However, the costs of FAW vary across countries and regions. Conway et al. (26) assessed the price of FAW in Australia at 5.61 USD/piece, based on the Australian Dollar (AUD) to US Dollar (USD) average exchange rate in 2022 (1 AUD = 0.6948 USD). In this study, we performed a cost analysis using the price of FAW in China, which could have led to inconsistent results.

The value of each minute of OR time was difficult to estimate and was affected by local costs and policies (27, 28). Studies have shown that the operating room costs in North America vary from 10 USD to 62 USD per minute in North America (28–31). However, few studies have estimated the cost of operating rooms in China. The average annual salaries of health practitioners in Beijing were obtained from the China Statistical Yearbook 2022. According to the Australian Bureau of Statistics, the average weekly earnings of staff in healthcare and social assistance in 2022 was 1225.34 USD, about 0.51 USD/min/person, which is nearly two times the staff costs in our study. The staff costs and the price of FAW blanket are crucial factors affecting the economic benefit of aggressive warming.

At present, hospitals in low-income and middle-income countries will face enormous financial pressure to take the initiative to provide aggressive warming, which ultimately leads to the difficulty of the wide use of aggressive warming and loss of thermal comfort for patients. We suggest health policies should be considered from the perspective of both patients and hospitals to encourage hospitals to provide better medical service. Under the current price system in China, compared to aggressive warming to 37°C, keeping intraoperative temperature at least 35.5°C might be equally safe and more economic. Aggressive warming can be used according to patient wishes.

In fact, availability and affordability to medical therapies remains one of the biggest health challenges faced by developing countries. The national volume-based procurement (NVBP) was launched in China aiming to reduce drug prices and improve the affordability of medicines and medical consumables (32), especially for patients with relatively low income. With the implementation of NVBP, healthcare costs for both hospitals and payers may be reduced, providing a potential solution for the major health challenges faced by all.

Our study had some limitations. First, the OR efficiency was analyzed based on the operating room regulation that no procedure could be performed after 18: 00 in our analysis, which would restrict the number of operations. Second, in some hospitals, extubation was achieved in the PACU, where reduced recovery time may not directly influence the OR efficiency or could cause decreased economic benefit because fewer staff members were affected, and this was not applicable to our study.

An additional limitation is that we did not assess the thermal comfort of patients using different thermal management strategies. Complications due to hypothermia, such as chills and feelings of weakness, can cause discomfort and uneasiness for patients but may have a slight effect on cost analysis.

However, our study provides a calculation method for the cost analysis of aggressive warming. The parameters in our analysis can be adjusted according to the reality of other hospitals or regions.

## 9. Conclusion

There was no significant difference in the total hospitalization costs between patients with and without aggressive intraoperative warming. From the perspective of a hospital, despite the decreased staff costs due to enhanced OR efficiency, its economic benefits depend on local hospital policies and the price of FAW, varying from different regions and countries.

## Data availability statement

The original contributions presented in the study are included in the article/Supplementary material, further inquiries can be directed to the corresponding author.

## Ethics statement

The studies involving humans were approved by Peking Union Medical College Hospital. The studies were conducted in accordance with the local legislation and institutional requirements. Written informed consent for participation in this study was provided by the participants’ legal guardians/next of kin.

## Author contributions

SS: Writing – original draft, Data curation. LP: Funding acquisition, Project administration, Writing – review & editing. HC: Methodology, Writing – review & editing. YZ: Methodology, Writing – review & editing. CS: Data curation, Writing – review & editing. JY: Writing – review & editing, Project administration. YH: Funding acquisition, Project administration, Writing – review & editing.

## Figures and Tables

**Table:** 

OR	operating room
DES	discrete event model
USD	US dollar
FAW	forced-air warming
PUMCH	Peking Union Medical College Hospital
AIMS	Anesthesia Information Management Systems
PACU	post anesthesia care unit
SD	standard deviation
LOS	length of stay
BMI	body mass index
HTN	hypertension
DM	diabetes mellitus
CRF	chronic renal failure
CAD	coronary artery disease
COPD	chronic obstructive pulmonary disease
PAD	peripheral arterial disease
SSI	organ/space surgical site infection
HAI	hospital-acquired Infection
MINS	myocardial injury after non-cardiac surgery
AUD	Australian dollar; NVBP: national volume-based procurement

## Footnotes

^1^ http://www.stats.gov.cn/sj/ndsj/2022/indexch.htm
