# Supplementary material for: Analysis of hospital and payer costs of care: aggressive warming versus routine warming in abdominal major surgery
Source: Front Public Health. 2023 Nov 2;11:1256254. doi: 10.3389/fpubh.2023.1256254 (PMC10652782; doi:10.3389/fpubh.2023.1256254)
Supplement: Supplementary file 1 [file Table_1.DOCX]

**Supplement 1** Estimated costs associated with the warming equipment

| Cost | | | Routine warming | Aggressive warming |
| --- | --- | --- | --- | --- |
| Equipment (USD) |  | |  |  |
| Direct cost | Medical supplies | | 4.46 | 74.34 |
|  | Forced-air warming blanket | | 0 | 74.34 |
|  | Routine quilt | | 4.46 | 0 |
|  | Energy cost | | 0 | 1.03 |
| Indirect cost | Medical equipment depreciation | | 0 | 2.88 |
|  | Repair and maintenance | | 0 | 0.43 |
| Overall |  | | 4.46 | 78.68 |
| Staff cost (USD/min/person) |  | 0.27 | | |
